# Hp1-1 as a Genetic Marker Regulating Inflammation and the Possibility of Developing Diabetic Complications in Patients with Type 2 Diabetes—Cohort Studies

**DOI:** 10.3390/genes11111253

**Published:** 2020-10-24

**Authors:** Anna Stempkowska, Magdalena Walicka, Edward Franek, Marek Naruszewicz, Mariusz Panczyk, Yaroslav Sanchak, Agnieszka Filipek

**Affiliations:** 1 Clinical Department of Internal Diseases, Endocrinology and Diabetology, Central Clinical Hospital of the MSWiA in Warsaw, Wołoska 137, 02-507 Warsaw, Poland; stempkowska.anna@gmail.com (A.S.); m_walicka@wp.pl (M.W.); Edward.franek@cskmswia.pl (E.F.); sanchaky@gmail.com (Y.S.); 2 Department of Pharmacognosy and Molecular Basis of Phytotherapy, Faculty of Pharmacy, Medical University of Warsaw, Banacha 1, 02-097 Warsaw, Poland; marek.naruszewicz47@gmail.com; 3 Department of Education and Research in Health Sciences, Faculty of Health Sciences, Medical University of Warsaw, Banacha 1, 02-097 Warsaw, Poland; mariusz.panczyk@wum.edu.pl

**Keywords:** haptoglobin phenotypes, inflammatory markers, type 2 diabetes, cardiovascular disease

## Abstract

Background: This study assessed the influence of the haptoglobin phenotype on markers regulating inflammation in patients with type 2 diabetes. Methods: The haptoglobin phenotypes, soluble form of CD163 receptor (sCD163), p53 concentrations and high mobility group box protein 1 (HMGB1), interleukin 10 (IL-10) secretion in serum were assayed via ELISA tests. In the first part of the project, patients were divided into three groups which differed by the haptoglobin phenotype, and afterwards into two groups according to the criterion of the presence or absence of cardiovascular disease. Results: Diabetic patients with haptoglobin phenotype 1-1 (Hp1-1) had a significantly higher concentration of IL-10 and sCD163 compared to haptoglobin phenotype 2-1 (Hp2-1) and haptoglobin phenotype 2-2 (Hp2-2). Moreover, diabetic patients with Hp1-1 had a significantly lower concentration of p53 and HMGB1 compared to diabetic patients with Hp2-1 and Hp2-2. The results have shown that diabetics with Hp2-1 had a significantly lower postprandial glucose level compared to diabetics with Hp2-2. Apart from that, there were no differences in the occurrence of haptoglobin variants between patients with or without cardiovascular disease. Conclusions: Our study provides new data for a relationship between the type of haptoglobin in patients with type 2 diabetes and the concentration of factors that regulate the body’s inflammation. We have shown that the Hp1-1 can serve as a genetic marker of inflammatory processes.

## 1. Introduction

The term “diabetes mellitus” defines a group of metabolic diseases characterized by hyperglycemia resulting from a defect in insulin secretion and/or action. Chronic hyperglycemia is associated with failure of various organs. One of the complications of diabetes that is a major cause of premature mortality in type 2 diabetes is cardiovascular disease [1].

Hyperglycemia has a toxic influence on the vascular endothelium, increases oxidative stress, inhibits the bioavailability of nitric oxide (NO) and creates advanced glycation-end products (AGEs) [2]. Insulin resistance, inherent in type 2 diabetes, potentiates atherosclerotic processes in arterial vessels and modifies the inflammatory processes in atherosclerosis. The evidence that the inflammatory process has a significant contribution to the mechanism of atherosclerosis is macrophage foam cells, which are the basic component of atherosclerotic plaques [3].

In diabetic patients, the arteries that become affected by atherosclerosis are characterized by increased neovascularization and blood vessel fragility. This leads to microhemorrhages and extravasation of erythrocytes, and the consequent release of extracorpuscular “free” hemoglobin (Hb) into the atherosclerotic plaque. Free hemoglobin is capable of producing reactive oxygen species [4].

Haptoglobin (Hp) is an acute-phase protein whose biological role is to bind free circulating hemoglobin (Hb) to produce a haptoglobin–hemoglobin (Hp–Hb) complex, thereby mediating its removal. Its affinity for free hemoglobin is an effective mechanism protecting tissues against the toxic effects of hemolysis [5]. This protein is produced by hepatocytes and occurs in the form of three phenotypes (haptoglobin phenotype 1-1 (Hp1-1), haptoglobin phenotype 2-1 (Hp2-1) and haptoglobin phenotype 2-2 (Hp2-2)) that differ in structure, biological properties and function [6].

Two polymorphic variants of haptoglobin (Hp1 and Hp2) and three phenotypes (Hp1-1, Hp2-1 and Hp2-2) have been identified in humans. It is known that haptoglobin is made up of four chains: two α and two β chains. Due to its low molecular weight (86 kDa), Hp1-1 has a high affinity for hemoglobin and can easily generate the haptoglobin–hemoglobin complexes. In contrast, the long chains Hp 2-2 (170–900 kDa) and Hp 2-1 (86–300 kDa) are difficult to connect to Hb and do not generate the haptoglobin–hemoglobin complexes. Therefore, it is suggested that the phenotype of Hp may be a marker for the development of cardiovascular complications in individuals who are suffering from diabetes. Specifically, it has been shown that people with diabetes and have the Hp2-2 phenotype are at a significantly increased risk of developing vascular complications, including stroke, myocardial infarction and kidney disease [7,8].

CD163 is a differentiation antigen of the monocyte–macrophage system, and with the highest expression of mature macrophages, it is related to anti-inflammatory type M2. It is known that in humans, CD163 receptor expression occurs in the acute phase of infection, in chronic inflammation, and in wound healing [9]. In vitro studies have shown that CD163 can be up-regulated by many factors including glucocortycosteroids, interleukin 10 (IL-10), and TGF-β. In contrast, pro-inflammatory mediators such as LPS, and TNF-α inhibit the expression of the CD163 receptor [10,11]. Changes in receptor expression as a result of anti-inflammatory and pro-inflammatory factors undoubtedly indicate a link to the body’s immune response. The physiological receptor function associated with the binding of Hp–Hb complexes is the best known to date. This receptor is involved in the clearance of hemoglobin–haptoglobin complexes by the resident macrophages and thus protects tissues against oxidative damage caused by free hemoglobin [12,13].

It is known that there is a soluble form of the CD163 receptor (sCD163). Oxidative stress mediators or lipopolysaccharides which appear during inflammation will facilitate the detachment of the soluble form of the CD163 receptor from the membrane form. On the other hand, inhibitors of metalloproteinases, e.g., TIMP-3, prevent the release of this receptor from the surface of monocytes–macrophages [14,15]. Current research results do not give a clear answer as to whether the plasma concentration of sCD163 reflects the activity of the CD163 receptor. The increased expression of the Toll-like receptor and the secretion of ADAMs in acute coronary syndrome are believed to inactivate the CD163 receptor, and, therefore, its soluble form does not appear in plasma [16]. On the other hand, Aristoteli et al. (2006) recommended sCD163 as a marker in coronary artery disease [17].

The activity of the CD163 receptor is directly dependent on the activity of the interleukin 10 receptor (IL-10). It is known that IL-10, secreted by lymphocytes of the Th2 subtype and activated macrophages, is an anti-inflammatory cytokine. IL-10 may greatly influence the local inflammatory process within the atherosclerotic lesion by acting as an antiatherogenic factor. In addition, through increasing the production of the tissue inhibitor of metalloproteinases-1 (TIMP-1) and inhibiting matrix metalloproteinase (MAP) synthesis, interleukin 1 may be involved in the stabilization of atherosclerotic plaques [18,19,20]. 

High mobility group box protein 1 (HMGB1) has a very broad spectrum of activity. Inside the cell, HMGB1 is involved in DNA replication, recombination, transcription and repair processes. Following release into the extracellular space, HMGB1 becomes a proinflammatory cytokine, and by associating with the RAGE receptor (receptor binding the glycation end products) or Toll-like membrane receptors (TLR 4 and 9), it initiates oxidative stress. HMGB1 is thought to be involved in cell apoptosis and necrosis as well as stimulating the proliferation and migration of M1 proinflammatory macrophages [21]. In atherosclerotic disease, HMGB1 shows increased expression in nuclei and cytoplasm of macrophages, as well as smooth muscle cells of atherosclerotic lesions. It is involved in the progression of the atherosclerotic plaque and amplification of the inflammatory response during acute ischemic injury [22]. In addition, the p53 protein (p53) is a transcription factor with tumor suppressor properties that plays a role in eliciting cellular responses to a variety of stress signals, including DNA damage, hypoxia and aberrant proliferative signals. When DNA repair fails, p53 induces cell apoptosis, thereby reducing the secretion of pro-inflammatory factors. It has been shown that both HMGB1 and p53 may increase the existing inflammation and contribute to the development of inflammatory diseases such as atherosclerosis and type 2 diabetes [23,24].

Due to the low survival rate of patients with type 2 diabetes after a cardiovascular incident and the fact that myocardial infarction or stroke of the central nervous system also affects people without known cardiovascular risk factors (dyslipidemia, nicotine use, hypertension), we decided to investigate the relationship between a specific haptoglobin phenotype and the activity of specific markers regulating inflammatory processes (SMRIP) in the body in patients with type 2 diabetes. Chronic inflammation may only be the beginning of the development of macrovascular complications in diabetes. Therefore, the aim of the study was also to assess whether the haptoglobin variant is associated with an increased risk of diabetic complications, including cardiovascular disease, in people with type 2 diabetes. Our research will help elucidate the role of genetic determinants and determine the function of a genetic marker that will predict which patients with type 2 diabetes will be at greater risk of developing complications.

## 2. Methods

### 2.1. Ethical Approval

This cohort study was conducted in accordance with the principles of the Helsinki Declaration. Ethical approval (number 8/2016) for this study was obtained from the Ethics Committee on Animal and Human Experimentation in the Central Clinical Hospital of the Ministry of the Interior and Administration in Warsaw. All subjects provided written informed consent to participate.

### 2.2. Study Population

The study included 94 patients with type 2 diabetes and coexisting obesity. Patients were hospitalized in the Department of Internal Diseases, Endocrinology and Diabetology of the Central Clinical Hospital of the Ministry of Interior and Administration in Warsaw or were outpatient of the Diabetes Center of the Clinic in 2016. A total of 56 men and 38 women between the ages of 40–75 years old were included. This group was included during self-monitoring of their blood glucose and had hyperglycemia above 180 mg/dL, and during metformin therapy in combination with insulin or another oral hypoglycemic medication. Exclusion criteria were active alcoholism, active cancer, a mental state preventing cooperation of the patient with the investigator, pregnancy and acute infection that could affect the metabolic control of diabetes.

### 2.3. Clinical and Biochemical Measurements

An interview about diabetes, its duration and past complications was conducted. In a prepared survey, the patient had to answer questions about the average fasting glycaemia and glycemia 2 h after meals, addiction to smoking, daily dose of metformin or other hypoglycemic medications. Anthropometric parameters were analyzed: age, sex, height, weight and waist circumference. BMI (body mass index) of study participants was calculated. Blood samples were taken and the concentration of parameters such as glycated hemoglobin (HbA1c), lipidogram, *C*-*reactive protein (CRP),* total calcium, phosphorus, vitamin D, and morphology were measured. In addition, albuminuria in the urine was assessed, fundus examinations and electrocardiograms were carried out, and exercise tests were performed to assess diabetes complications in selected patients.

### 2.4. Haptoglobin Phenotyping

In our work, we determined the haptoglobin phenotype in patients using an enzyme-linked immunosorbent assay (Haptoglobin (Hp) ELISA, Technion-Israel Institute of Technology, Haifa, Israel) according to the manufacturer’s procedure. The result was the average of three measurements for each patient. The values of Optical Density (OD) at 450 nm of each supernatant determined the Hp types: Hp1-1~0.495, Hp2-1~1.382, and Hp2-2~1.819. On this basis, patients were assigned to the group Hp1-1, Hp2-1 or Hp2-2.

Additionally, using the Hardy–Weinberg principle, the incidence of haptoglobin phenotypes in the population of patients with type 2 diabetes and the general population was calculated.

### 2.5. Assessment of Serum sCD163 Concentration

It is known that the CD163 scavenger receptor (130 kDa) is a biomarker of anti-inflammatory M2 macrophages and a down-regulator of the inflammatory response. Moreover, the CD163 receptor shows high affinity for haptoglobin–hemoglobin complexes in order to remove erythrocytes-derived free hemoglobin [9]. Current research acknowledges a protective role of CD163 expression in atherosclerotic tissue, where they can limit pathogenic effects of intraplaque hemorrhages such as plaque rupture by removing free hemoglobin and switch macrophages from the proinflammatory type M1 to the anti-inflammatory type M2 [8,25].

The soluble form of CD163, noted as sCD163, is present in the plasma of healthy individuals and is a reflection of CD163 receptor activity. Many studies suggest that sCD163 may be a sensitive biomarker of inflammation, and increased levels have been found in numerous pro-inflammatory conditions, i.e., sepsis, hepatitis, rheumatoid arthritis, scleroderma or atherosclerosis [26].

The levels of sCD163 in serum were determined by an enzyme-linked immunosorbent assay (Human CD163 Quantikine ELISA Kit, R&D System, Abingdon, UK) according to the manufacturer’s procedure. Results are reported as ng of sCD163 per mL of serum.

### 2.6. Assessment of Serum IL-10 Concentration

Interleukin 10 (IL-10) as an anti-inflammatory cytokine is necessary to maintain a balance between pro-inflammatory and anti-inflammatory processes. It is known that IL-10 is strongly associated with CD163 receptor expression. The binding between haptoglobin and hemoglobin by induction of the CD163 receptor is stimulated by enhanced IL-10 secretion [8,27].

The levels of IL-10 in serum were determined by an enzyme-linked immunosorbent assay (Human IL-10 Quantikine ELISA Kit, R&D System, Abingdon, UK) according to the manufacturer’s procedure. Results are reported as pg of IL-10 per mL of serum.

### 2.7. Assessment of Serum p53 Extracellular Secretion

The p53 protein is involved in the regulation of many cellular processes, in particular the activation of DNA repair mechanisms or the induction of apoptosis in response to DNA damage. In a healthy cell, p53 is inactive and its serum levels are negligible. Activation of the p53 protein involves its phosphorylation and may be associated with the intensifies of apoptosis in the event of irreversible cell damage [28].

The levels of p53 in serum were determined by an enzyme-linked immunosorbent assay (Human p53 ELISA, RayBiotech, Peachtree Corners, GA, USA) according to the manufacturer’s procedure. Results are reported as ng of p53 per mL of serum.

### 2.8. Assessment of Serum HMGB1 Extracellular Secretion

HMGB is secreted by immune cells, such as macrophages, monocytes and dendritic cells. It is thought that extracellular HMGB1 is a marker of cell ischemia and damage. It is known that HMGB1 plays multiple roles in the pathogenesis of inflammatory diseases [29].

Extracellular HMGB1 levels were determined by an enzyme-linked immunosorbent assay (Human HMGB1/ HMG-1 Quantikine ELISA Kit, R&D System, Abingdon, UK) according to the manufacturer’s procedure. Results are reported as pg of HMGB1 per mL of serum.

### 2.9. Statistical Analyses

In the first part of the project, patients were divided into three groups differing in haptoglobin phenotype (Hp1-1. Hp 2-1, Hp 2-2). The median (Mdn) and the interquartile range (IQR) were used to describe the quantitative variables. Qualitative variables were presented using the number (N) and the percent (%). The Fisher–Freeman–Halton exact test was applied to compare the qualitative variables. The quantitative variables in three groups were compared using Kruskal–Wallis rank test. Since a normal distribution was not observed and study groups (*N* = 9, *N* = 37, *N* = 48) were of unequal sizes, non-parametric statistics were used. In all cases, when this test showed statistically significant differences (rejection of the H0 hypothesis), Dunn’s post hoc test was used to identify significant differences for individual pairs (multiple rank average comparison test). Effect size was estimated with the use of eta-squared (*η*^2^). The following criteria were assumed to assess the measured effect size: large ≥ 0.140, intermediate 0.059–0.139, small 0.010–0.059, and no effect < 0.01. The effects with a probability value (*p*) lower than the significance level of 0.05 (*p* < 0.05) were assumed significant. Statistical analysis was performed in the TIBCO^®^ Software Statistica™ version 13.3 (Palo Alto, CA, USA).

In the second part of the study, the same group of 94 patients was divided according to the criterion of presence or absence of cardiovascular disease (the control group included patients without cardiovascular disease). Due to the fact that the sizes of the compared groups were less than 100 (*N* = 60, *N* = 34) and additionally that the sizes were not equal, it was necessary to approach the possibility of using parametric tests very restrictively. The most important assumption was tested, i.e., compliance with the normal distribution of individual variables in the tested groups (Shapiro–Wilk test). Normal distribution was observed for only 8 quantitative variables. For these variables, parametric analysis by Student’s t-test could be used. For the remaining quantitative variables, the non-parametric Mann–Whitney U test was used.

## 3. Results

### 3.1. Haptoglobin Phenotyping

Immunoenzymatic analysis showed that in the studied group of people with type 2 diabetes, nine individuals had the Hp1-1 phenotype, 37 had the Hp 2-1 phenotype and 48 had the Hp 2-2 phenotype. Based on the obtained results, we calculated the incidence of haptoglobin phenotypes in the population of patients with type 2 diabetes. In the current study, the most common Hp allele in patients with type 2 diabetes was Hp 2 (0.71). The frequency of the Hp1 allele was 0.29.

### 3.2. Comparative Analysis Results According to the Criterion of the Haptoglobin Phenotype

The influence of the haptoglobin phenotype on the concentration of specific markers (sCD163, IL-10, HMGB1 and p53) regulating inflammatory processes in the body in patients with type 2 diabetes was determined. Moreover, we assessed the relationship between the haptoglobin phenotype and individual clinical parameters in the same patients.

**Figure 1:**
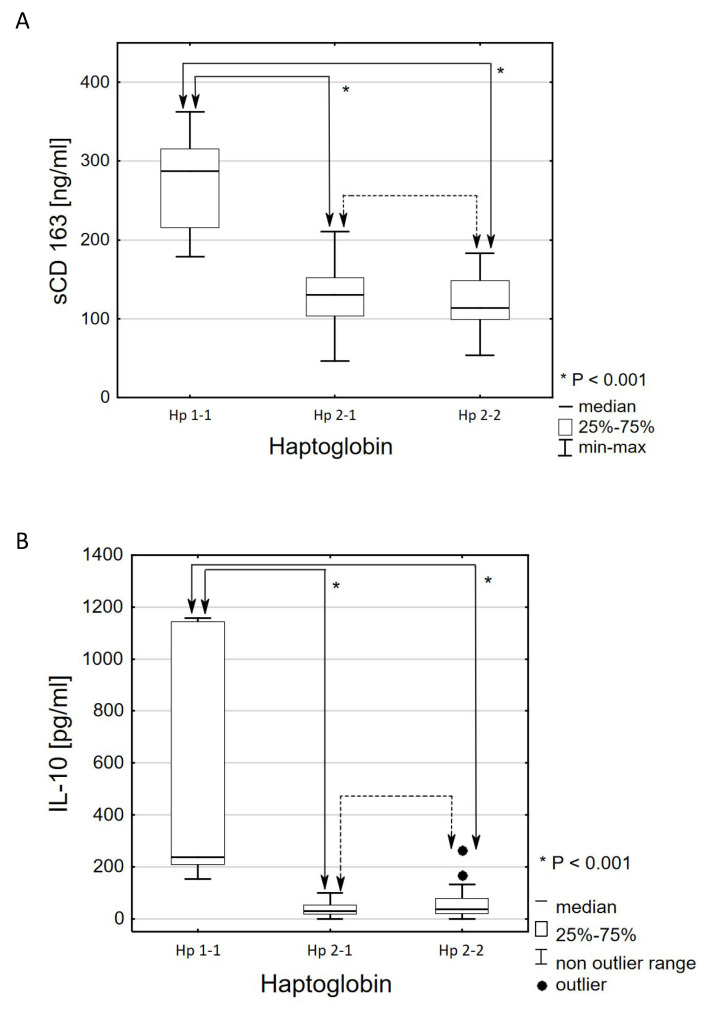
Effect of haptoglobin phenotype on serum soluble form of CD163 receptor (sCD163) (**A**) and interleukin 10 (IL-10) (**B**) concentration in patients with type 2 diabetes. The sCD163 concentration is expressed as ng/mL serum ± interquartile range (IQR). The IL-10 concentration is expressed as pg/mL serum ± IQR. * *p* < 0.001—statistical significance between Hp1-1 (control group) and Hp2-1 or Hp2-2. *p* > 0.05—no statistically significant differences between Hp2-1 and Hp2-2. Hp1-1—haptoglobin phenotype 1-1; Hp2-1—haptoglobin phenotype 2-1; Hp2-2—haptoglobin phenotype 2-2.

The haptoglobin variant in a patient with type 2 diabetes had a significant impact on the concentration of all four activities of SMRIP. Patients with haptoglobin type 1-1 had statistically significantly higher level of sCD163 than patients with haptoglobin type 2-1 (Mdn: 287.4 ± 99.7 ng/mL vs. 130.4 ± 48.3 ng/mL; *p* < 0.001) and patients with type haptoglobin 2-2 (Mdn: 287.4 ± 99.7 ng/mL vs. 114.0 ± 48.6 ng/mL; *p* < 0.001) (Figure 1A).

Similar relationships were demonstrated by comparing the results of interleukin 10. It was found that the haptoglobin variant had a significant effect on the concentration of IL-10 (*p* < 0.001, *η*^2^ = 0.245). Patients with haptoglobin type 1-1 had statistically significantly higher concentrations of IL-10 than patients with haptoglobin type 2-1 (Mdn: 236.4 ± 933.7 pg/mL vs. 30.4 ± 35 pg/mL; *p* < 0.001) and patients with haptoglobin type 2-2 (Mdn: 236.4 ± 933.7 pg/mL vs. 35.8 ± 58.5 pg/mL; *p* < 0.001) (Figure 1A).

**Figure 2:**
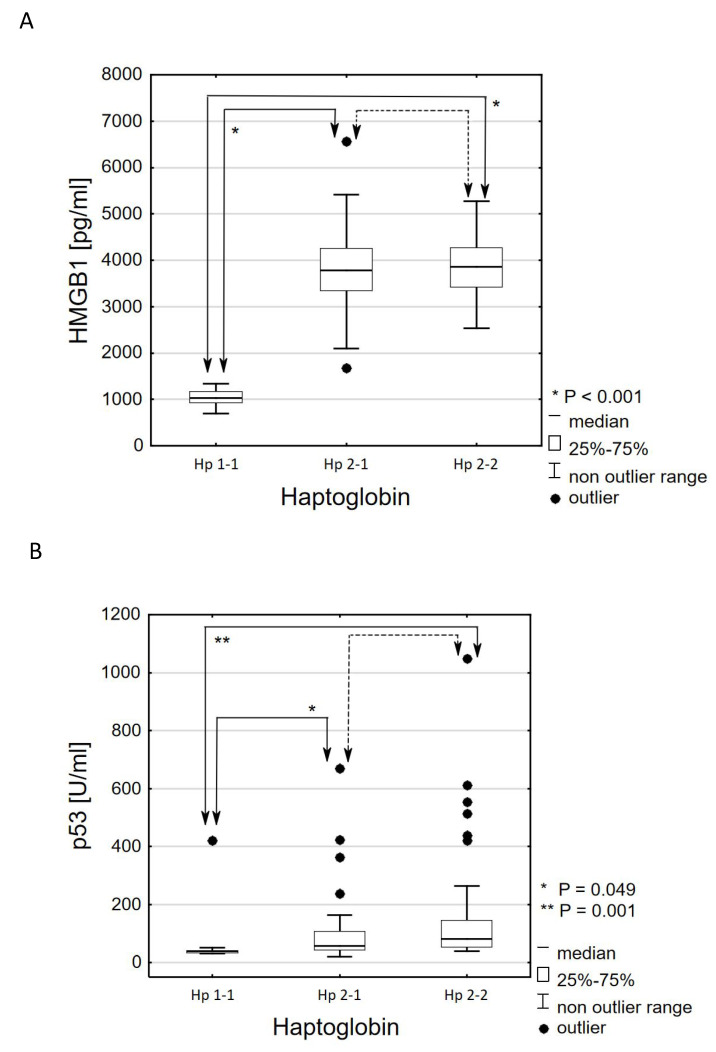
Effect of haptoglobin phenotype on serum high mobility group box protein 1 (HMGB1) (**A**) and p53 (**B**) extracellular secretion in patients with type 2 diabetes. The HMGB1 extracellular secretion is expressed as pg/mL serum ± IQR. * *p* < 0.001—statistical significance between Hp1-1 (control group) and Hp2-1 or Hp2-2. *p* > 0.05—no statistically significant differences between Hp2-1 and Hp2-2. The p53 extracellular secretion is expressed as U/mL serum ± IQR. * *p* = 0.049, ** *p* = 0.001—statistical significance between Hp1-1 (control group) and Hp2-1 or Hp2-2. *p* > 0.05—no statistically significant differences between Hp2-1 and Hp2-2. Hp1-1—haptoglobin phenotype 1-1; Hp2-1—haptoglobin phenotype 2-1; Hp2-2—haptoglobin phenotype 2-2.

The effect of the haptoglobin variant also turned out to be statistically significant on the concentrations of pro-inflammatory markers. Patients with type haptoglobin 1-1 had statistically significantly lower levels of HMGB1 secretion than patients with haptoglobin type 2-1 (Mdn: 1024.5 ± 244.5 pg/mL vs. 3786.5 ± 903.6 pg/mL; *p* < 0.001) and patients with type haptoglobin 2-2 (Mdn: 1024.5 ± 244.5 pg/mL vs. 3585.2 ± 840.6 pg/mL; *p* < 0.001) (Figure 2A). Patients with haptoglobin type 1-1 also had significantly lower levels of p53 secretion than patients with type 2-1 haptoglobin (Mdn: 39.1 ± 7.1 U/mL vs. 57.5 ± 64.6 U/mL; *p* = 0.049) and patients with type haptoglobin 2-2 (Mdn: 39.1 ± 7.1 U/mL vs. 81.6 ± 91.9 U/mL; *p* = 0.001) (Figure 2B).

All detailed results are included in the Supplementary materials (Both Table S1 and S2).

### 3.3. Comparative Analysis Results According to the Criterion of the Presence or Absence of Cardiovascular Disease

In this part of the work, the relationship between individual clinical parameters, haptoglobin phenotypes and concentrations of SMRIP and the presence or absence of cardiovascular disease in patients with type 2 diabetes was analyzed. There were no statistically significant differences in the occurrence of haptoglobin variants between patients with or without cardiovascular disease (Table 1). There were also no statistically significant differences in the concentrations of specific markers regulating inflammatory processes (Table 2). However, among all the clinical parameters examined, only a few showed statistically significant differences (Table 2 and Table 3). Patients with cardiovascular complications were characterized by a statistically significantly longer duration of the disease than patients from the group without such complications (Mdn: 15.0 ± 10 years vs. 10.0 ± 10 years; *p* = 0.003), as well as a statistically significantly higher postprandial glucose level than patients from the group without such complications (Mdn: 250.0 ± 100 mg/dL vs. 205.0 ± 90 mg/dL; *p* = 0.039) (Table 2). Higher levels of RDW-SD (red blood cell distribution width standard deviation) have also been shown in people with cardiovascular complications (Mdn: 44.3 ± 5.2 vs. 42.0 ± 3.3; *p* = 0.038) (Table 2).

The differences for the analyzed lipidogram parameters were also statistically significant. In the group of patients not taking lipid-lowering drugs, people with cardiovascular complications were characterized by a statistically significantly lower concentration of High-density lipoprotein (HDL) cholesterol (Mdn: 42.0 ± 8 mg/dL vs. 46.5 ± 11.5 mg/dL; *p* = 0.044; *η*^2^ = 0.135) and a statistically significantly higher concentration of Low-density lipoprotein (LDL) cholesterol (Mdn: 134.0 ± 59 mg/dL vs. 86.0 ± 41 mg/dL; *p* = 0.006; *η*^2^ = 0.241), total cholesterol (Mdn: 226.0 ± 62 mg/dL vs. 160.5 ± 42.5 mg/dL; *p* = 0.015; *η*^2^ = 0.195) and triglyceride concentration (Mdn: 190.0 ± 255 mg/dL vs. 108.5 ± 73.5 mg/dL; *p* = 0.006; *η*^2^ = 0.245) (Table 4). In the group of patients receiving lipid-lowering drugs, patients with cardiovascular complications were in turn characterized by statistically significantly lower concentration of LDL cholesterol than patients from the group without such complications (Mdn: 64.0 ± 41 mg/dL vs. 108.5 ± 58 mg/dL; *p* = 0.001; *η*^2^ = 0.167) and a statistically significantly lower concentration of total cholesterol (Mdn: 135.0 ± 40 mg/dL vs. 186.5 ± 60.5 mg/dL; *p* < 0.001; *η*^2^ = 0.189) (Table 4).

## 4. Discussion

In our case–control study, we demonstrated for the first time an association between the haptoglobin phenotype and the concentration of specific markers (sCD163, IL-10, HMGB1 and p53) regulating inflammatory processes in the body in patients with type 2 diabetes. People with the Hp 2-2 phenotype had significantly higher concentration of pro-inflammatory markers and lower concentration of anti-inflammatory markers in the serum compared to people with Hp2-1 or Hp1-1 phenotypes. 

In the study, we have shown that patients with haptoglobin 1-1 had statistically significantly higher concentration of sCD163 and IL-10 than patients with haptoglobin type 2-1 and patients with type haptoglobin 2-2. It is known that under physiological conditions, the concentration of sCD163 in the blood remains at a fairly high level, on average about 2 mg/L [9]. Hence, possible statistically significant differences between Hp1-1 and Hp2-1, Hp2-2 exist. On the other hand, the reduction in blood sCD163 concentration (patients with Hp2-1 and Hp2-2) may be due to both an increase in CD163 expression and a decrease in CD163 synthesis. The Hp 2-2 phenotype is associated with an increase in oxidative stress and inflammatory processes that may lead to increased expression of the CD163 receptor. However, protease receptors present under these pathological conditions may prevent sCD163 from being released from the surface of monocytes-macrophages [14,15]. It is also noteworthy that the increase in CD receptor expression is associated with IL-10. Levy et al. (2007) showed that the binding of Hp1-1 complexes to haptoglobin enhances the production of IL-10, which is known to upregulate the CD163 receptor [4]. Our research confirmed that people with Hp1-1 had significantly higher levels of IL-10 in the blood than patients with Hp2-1 and Hp2-2.

When talking about sCD163, its correlation with the CD163 receptor should also be taken into account. Levy et al. (2007) and Guetta et al. (2007) showed an inverse correlation between sCD163 and CD163 [4,30]. Our research, however, suggests that the concentration of sCD163 is closely related to the expression of the CD163 receptor. Inhibition of CD163 expression by a number of receptor-related factors (inactivation of IL-10, Toll Like receptors, ADAMs) may significantly decrease sCD163 levels [16].

For the first time, our research showed the dependence of the haptoglobin phenotype on the concentration of specific pro-inflammatory markers in the blood in patients with type 2 diabetes. Individuals with type haptoglobin 1-1 had statistically significantly lower concentrations of HMGB1 and p53 than patients with type 2-1 and patients with type haptoglobin 2-2. 

The pleiotropic effects of p53 in patients with type 2 diabetic are controversial. There are several studies showing that p53 may inhibit diabetes. The protective role of p53 in diabetes is believed to be related to the p53 codon 72 polymorphism and the arginine 72 variant of p53 in humans and in an animal model [31,32]. However, far more reports prove that p53 activity contributes to the development of diabetes or the development of complications in people with type 2 diabetes [33]. P53 is known to be an intracellular protein and is responsible for the DNA repair processes in the cell. When DNA repair fails, p53 induces cell cycle arrest, senescence, and programmed cell death. Therefore, it seems that the appearance of certain amounts of extracellular p53 may indicate increased apoptosis and necrosis of cells. Additionally, p53-dependent cell death is associated with the production of pro-inflammatory factors, and their overproduction causes chronic inflammation in the body and the development of metabolic diseases, including type 2 diabetes [23]. Studies in an animal model (Lig4^−/−^; p53^−/−^ mice) have confirmed that p53-mediated aging of adipocytes and p53-mediated pancreatic β cells induces the development of insulin resistance and diabetes [34]. In turn, Zang et al. (2013, 2014) suggests that non-tumor-associated forms of p53 inhibit the translocation of glucose transporters (GLUT1, GLUT4) to the plasma membranes, overexpression RRAD (Ras-related associated with diabetes), which impairs glucose uptake and results in insulin resistance in muscle and adipose tissues [35,36]. It is also worth noting that p53 affects glucose levels by negatively regulating glycolysis and positively regulating gluconeogenesis. As is known, these processes can lead to the gradual use of insulin secretory reserves and the breakdown of glucose metabolism [33]. Li et al. (2018) confirmed that high glucose levels induce apoptosis in retinal endothelial cells by activating the p53-dependent signaling pathway [37].

To prove the role of protein p53 in the development of other inflammatory diseases, Kolovou et al. (2018) evaluated the relationship between gene polymorphism of p53 and ischemic heart disease in his study, and Chang et al. (2013) suggested a link between post-translational modification of p53 and ERK5 proteins detected during endothelial apoptosis and inflammation which contributes to the formation of atherosclerotic plaque [24,38].

HMGB1, as a non-histone nuclear protein regulating gene expression, is an endogenous signaling molecule that triggers inflammatory responses when released into the extracellular environment. It is known that extracellular HMGB1 is a biomarker of cell ischemia and cell damage. High levels of HMGB1 have been observed in diabetics with diabetic nephropathies and retinopathy and insulin resistance [39,40,41]. Moreover, a correlation between HMGB1 levels and diabetes complications has been found in both animal models and in humans with type 2 diabetes. In vivo studies, Hagiwara et al. (2008) showed that glucose infusion-induced hyperglycemia in rats was associated with elevated serum HMGB1 levels [42]. Similar results were obtained by Dasu et al. (2010) and Škrha et al. (2012) in people with hyperglycemia [43,44]. Elevated levels of HMGB1 have also been found in other tissues, such as adipose tissue, liver and muscle, in diabetic patients and in animal models. Other studies have shown that extracellular HMGB1 enhances pancreatic β-cell apoptosis. In turn, islet necrotic β-cells release HMGB1 to accelerate cell damage, creating a vicious loop. Moreover, the interaction among HMGB1, RAGE and TLR further exacerbates inflammation and increases the development of complications in patients with type 2 diabetes [40].

The role of HMGB1 in the development of atherosclerosis has been demonstrated in mice deficient in apolipoprotein E, which were fed high-fat products. Giving the mice a neutralizing monoclonal antibodies against HMGB1 reduced the development of atherosclerosis by 55% [45]. In addition, HMGB1 may be biomarker that allows for the prediction of the risk of cardiovascular death in patients with acute coronary syndrome [46]. It is known that an unstable atherosclerotic plaque is associated with an imbalance between anti-inflammatory (M2) and pro-inflammatory (M1) macrophages, to the benefit of the latter. M1 macrophages form the core of the atherosclerotic plaque, produce pro-inflammatory cytokines (TNF-α, IL-1β, IL-6), increasing the existing inflammation, which in turn leads to the microhemorrhage and destabilization of the atherosclerotic plaque [47]. M2 macrophages, which are activated by IL-10 and the expression of the CD163 receptor, produce anti-inflammatory factors (TGF-β, IL-10) that facilitate tissue remodeling and contribute to the stabilization of atherosclerotic plaque.

From the above data, it is known that the haptoglobin 1-1 phenotype lowers the level of pro-inflammatory factors and increases the level of anti-inflammatory factors in the blood of people with type 2 diabetes. Thus, it may be a genetic marker that predicts which patients are more likely to develop diabetic complications.

**Figure 3:**
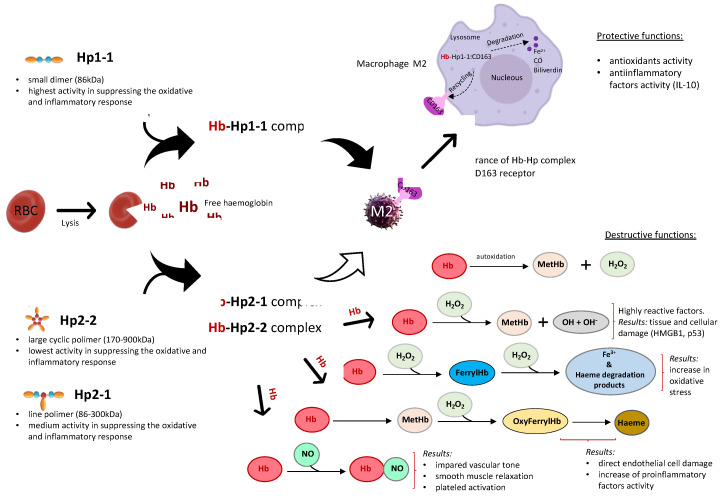
The proposed biological mechanism explaining the relationship between the haptoglobin phenotype and specific markers regulating inflammatory processes. In individuals with type 2 diabetic and Hp1-1 phenotype haptoglobin, hemoglobin (Hb) released intravascularly from red blood cells (RBC) is rapidly bound by haptoglobin (Hp1-1) protein to form an Hb–Hp1-1 complex that is cleared by scavenger receptor CD163 (on type anti-inflammatory macrophage M2). As a result, we obtain a protective effect through the increased production of anti-inflammatory (IL-10) and antioxidant factors. In people with type 2 diabetes and haptoglobin with the Hp2-1 and Hp2-2 phenotype, CD163 clearance is impaired and leads to increased oxidative stress. This results in an enhanced production of inflammatory factors which lead to destruction of the body’s tissues by apoptosis and necrosis of cells. It is known that chronic oxidative stress and the activity of pro-inflammatory factors may intensify the development of complications in people with type 2 diabetes, such as retinopathy, nephropathy, ischemic heart disease, atherosclerosis, stroke, and heart attack.

In most western populations, the most common haptoglobin phenotype is Hp 2-1 (48%). The incidence of Hp 2-2 is 36%, while Hp1-1 is only 16%. The distribution is different in some geographical regions, such as, for instance, Southeast Asia, where 90% of the population are people with the Hp 2-2 phenotype [48]. Comparing the data for the European population presented in the study of de Albuquerque Wobeto et al. (2017) with our own data, the frequency of the Hp1 allele in patients in our study is clearly lower than in the majority of European countries tested (except for Italy) (Hp1 allele 0.29 for our study vs. 0.4 for Belgium, 0.39 for Germany vs. 0.35 for Greece vs. 0.35 for Hungary vs. 0.35 for Moldova vs. 0.38 for Norway vs. 0.38 for Spain vs. 0.41 for Great Britain vs. 0.11 for Italy) [49]. Our results indicate that people with type 2 diabetes and haptoglobin 2-1 or 2-2 variant have higher levels of selected pro-inflammatory markers and lower anti-inflammatory markers. It follows that haptoglobin 2 alleles have a higher risk of developing the disease. The studies of Spanish or Chinese population have led to similar conclusions [50,51]. They also showed that inherited factor such as the phenotype of haptoglobin may play an important role in the pathogenesis of type 2 diabetes. Individuals with Hp 2-2 may have higher risk of type 2 diabetes because of the functional differences among the Hp phenotypes. This could have important clinical consequences. The proposed biological mechanism explaining the relationship between the haptoglobin phenotype and specific markers regulating inflammatory processes is presented in Figure 3.

Unfortunately, in the present study, we did not notice any significant differences in the occurrence of haptoglobin variants between patients with or without cardiovascular disease. There were also no statistically significant differences in the concentrations of individual factors regulating the body’s defense mechanisms between these two groups. This is possibly related to the fact that our study was an observational design on a random sample of the population with type 2 diabetes. We did not excluded participants with prior stroke or myocardial infarction—there were patients after acute cardiovascular incident and without such complications in the study. The diagnosis of a cardiovascular disease was based primarily on a questionnaire, exercise tests or echocardiography. We did not use coronary angiography or tomography of the coronary arteries in this study to confirm cardiovascular disease. However, it is known that other research on the impact of haptoglobin gene polymorphism on cardiovascular complications in diabetics has shown conflicting results. There have been reports in the literature of both an association between cardiovascular risk with the haptoglobin 2 [52,53,54,55,56] and haptoglobin 1 [5,57] alleles as well as a lack of such a relationship in both cases [5,58,59].

## 5. Conclusions

Our study provides new evidence for the relationship between the type of haptoglobin in a patient with type 2 diabetes and the concentration of SMRIP. It has been proven that the haptoglobin 1-1 phenotype significantly reduces the pro-inflammatory factors (HMGB1, p53) and increases the concentration of anti-inflammatory factors (sCD163, IL-10) in the blood. It therefore appears that a type of haptoglobin phenotype 1-1 can be considered a genetic marker of inflammation. Moreover, it appears that IL-10 may be an independent marker of disease in people with type 2 diabetes.

Unfortunately, no significant association has been found between the haptoglobin variant and an already established cardiovascular disease. However, we believe that haptoglobin phenotyping in people with type 2 diabetes may identify people with an increased risk of diabetic complications, including cardiovascular disease. However, more research is needed to understand the mechanism of the relationship between haptoglobin alleles and cardiovascular risk.

## Figures and Tables

**Table 1:** Classification of physical chemistry parameters in patients with type 2 diabetes due to cardiovascular complications. Frequency analysis of quantitative variables. Parametric analysis with the t-student test.

Variable	Group without Complications (*N* = 60)		Group with Complications (*N* = 34)		t_(df = 92)_	*p*-Value ^(1)^
	M	SD	M	SD		
Age(years)	59.08	7.60	61.88	6.97	−1.767	0.081
Waist (cm)	111.32	15.23	116.03	11.58	−1.564	0.121
HbA1c (%)	9.35	1.62	9.41	1.39	−0.188	0.851
Phosphorus (mg/dL)	3.63	0.65	3.79	0.58	−1.186	0.239
Neutrophils (%)	56.21	7.83	55.59	6.79	0.390	0.698
Lymphocytes (k/uL)	2.31	0.69	2.38	0.57	−0.496	0.621
MCV (fl)	89.81	3.78	91.21	4.62	−1.599	0.113
Platelets (k/uL)	239.77	60.17	237.32	50.14	0.200	0.842

M—mean, SD—standard deviation, df —degrees of freedom. ^(1)^ Student’s t-test. HbA1c—Glycated haemoglobin, MCV—Mean Corpuscular Volume.

**Table 2:** Classification of physical chemistry parameters in patients with type 2 diabetes due to cardiovascular complications. Frequency analysis of quantitative variables. Non-parametric analysis with the Mann–Whitney U test.

Variable	Group without Complications (*N* = 60)		Group with Complications (*N* = 34)		z	*p*-Value ^(2)^
	Mdn	IQR	Mdn	IQR		
BMI (kg/m^2^)	32.0	8.5	35.0	8.0	−1.502	0.13
Duration of diabetes (years)	10.0	10.0	15.0	10.0	−2.994	0.003
Fasting glucose (mg/dL)	150.0	60.0	175.0	50.0	−1.707	0.090
Glycemia 2 h after a meal (mg/dL)	205.0	90.0	250.0	100.0	−2.090	0.039
CRP (mg/L)	2.2	2.6	2.7	3.5	−0.709	0.478
Ca (mmol/L)	2.4	0.1	2.4	0.2	−0.559	0.576
Vitamin D (ng/mL)	16.8	9.9	18.5	9.1	0.354	0.722
Daily dose of metformin (mg)	2550.0	1225.0	2550.0	1500.0	0.562	0.586
Leukocytes (tys./uL)	7.1	2.4	7.6	1.8	−0.826	0.408
Neutrophils (tys./uL)	3.9	2.0	3.9	1.2	−0.378	0.704
Lymphocytes (%)	30.5	9.8	31.6	8.2	−0.956	0.341
Hemoglobin (g/dL)	14.6	1.9	13.6	2.3	1.583	0.113
Erythrocytes (mln/uL)	4.9	0.6	4.8	0.8	0.775	0.440
Hematocrit (%)	44.3	5.7	42.7	6.5	1.165	0.244
RDW-SD	42.0	3.3	44.3	5.2	−2.062	0.038
RDW (%)	12.9	1.1	13.1	1.0	−1.611	0.108
Il−10 (pg/mL)	31.9	43.7	46.1	66.0	−1.319	0.188
Protein p53 (U/mL)	62.4	66.8	60.9	110.2	−0.574	0.565
sCD163 (ng/mL)	118.0	58.3	135.1	62.1	−1.098	0.274
HMGB1 (pg/mL)	3811.5	834.1	3416.2	1346.8	1.432	0.152

Mdn—median, IQR—interquartile range. ^(2)^ Mann–Whitney U test. BMI—Body Mass Index, CRP—C Reactive Protein, RDW-SD—Red Cell Distribution Width Standard Deviation, sCD163—soluble form of CD163.

**Table 3:** Classification of physical chemistry parameters in patients with type 2 diabetes due to cardiovascular complications. Frequency analysis of quantitative variables. Non-parametric analysis with the Mann–Whitney U test.

Variable	Variants	Group without Complications (*N* = 60)		Group with Complications (*N* = 34)		*p*-Value
		N	%	N	%	
Sex	Men	37	61.7	19	55.9	0.664 ^(3)^
	Women	23	38.3	15	44.1	
Nicotine addiction	No	32	53.3	12	35.3	0.132 ^(3)^
	Yes	28	46.7	22	64.7	
Retinopathy	No	52	86.7	27	79.4	0.389 ^(3)^
	Yes	8	13.3	7	20.6	
Additional treatment	Insulin	40	66.7	27	79.4	0.239 ^(3)^
	Oral medications	20	33.3	7	20.6	
Statins or fibrates	No	20	33.3	9	26.5	0.643 ^(3)^
	Yes	40	66.7	25	73.5	
Haptoglobin	Hp1-1	5	8.3	4	11.8	0.569 ^(4)^
	Hp2-1	26	43.3	11	32.4	
	Hp2-2	29	48.3	19	55.9	

^(3)^ Fisher’s exact test two-sided ^(4)^ Fisher–Freeman–Halton exact test.

**Table 4:** Classification of lipidogram parameters in patients with type 2 diabetes due to cardiovascular complications. Frequency analysis of quantitative variables. Parametric analysis with the U Mann–Whitney test.

Variable	Group without Complications		Group with Complications		z	*p* -Value ^(5)^	*η^2^* ^(6)^
	Mdn	IQR	Mdn	IQR			
Group not taking lipid-lowering drugs (3 vs. 11 vs. 15)							
HDL (mg/dL)	46.5	11.5	42.0	8.0	1.982	0.044	0.135
LDL (mg/dL)	86.0	41.0	134.0	59.0	-2.641	0.006	0.241
Total cholesterol	160.5	42.5	226.0	62.0	−2.381	0.015	0.195
TG (mg/dL)	108.5	73.5	190.0	255.0	−2.664	0.006	0.245
Group taking lipid-lowering drugs (40 vs. 25)							
HDL (mg/dL)	41.5	16.5	43.0	10.0	−0.270	0.784	0.001
LDL (mg/dL)	108.5	58.0	64.0	41.0	3.291	0.001	0.167
Total cholesterol	186.0	60.5	135.0	40.0	3.506	0.000	0.189
TG (mg/dL)	154.0	134.5	148.0	74.0	0.930	0.351	0.013

Mdn—median, IQR—interquartile range, ^(5)^ U Mann–Whitney test, ^(6)^ the size of the effect. HDL—High-density lipoprotein, LDL—Low-density lipoprotein, TG—Triglycerides.

## Supplementary Materials

The following are available online at https://www.mdpi.com/2073-4425/11/11/1253/s1, Table S1: Frequency analysis of quantitative variables. Classification due to haptoglobin type. Table S2: Frequency analysis of quantitative variables. Parametric analysis with the t-student test Classification due to cardiovascular complications.

## Abbreviations

SMRIP	specific markers regulating inflammatory processes
Hp	haptoglobin
Hp1-1	haptoglobin phenotype 1-1
Hp2-1	haptoglobin phenotype 2-1
Hp2-2	haptoglobin phenotype 2-2
HMGB1	high mobility group box protein 1
IL-10	interleukin 10
sCD163	soluble form of CD163 receptor
RDW-SD	red blood cell distribution width
