# Supplementary material for: Hp1-1 as a Genetic Marker Regulating Inflammation and the Possibility of Developing Diabetic Complications in Patients with Type 2 Diabetes—Cohort Studies
Source: Genes (Basel). 2020 Oct 24;11(11):1253. doi: 10.3390/genes11111253 (PMC7716206; doi:10.3390/genes11111253)
Supplement: Supplementary file 1 [file genes-11-01253-s001.pdf]

Supplementary materials S1

Hp1-1 as a genetic marker regulating inflammation and the possibility of developing diabetic complications in patients with type 2 diabetes – cohort studies.

Stempkowska A.[1], Walicka M. [1], Franek E.[1], Naruszewicz M.[2], Panczyk M. [3], Filipek A.[2*]

[1] Clinical Department of Internal Diseases, Endocrinology and Diabetology, Central Clinical Hospital of the MSWiA in Warsaw, Wołoska 137, 02-507 Warsaw, Poland

[2] Department of Pharmacognosy and Molecular Basis of Phytotherapy, Faculty of Pharmacy. Medical University of Warsaw, Banacha 1, 02-097 Warsaw, Poland

[3] Department of Education and Research in Health Sciences. Faculty of Health Sciences. Medical University of Warsaw, Banacha 1, 02-097 Warsaw, Poland

* Corresponding author: Department of Pharmacognosy and Molecular Basis of Phytotherapy, Faculty of Pharmacy, Medical University of Warsaw, Banacha 1, 02-097 Warsaw, Poland

Tel./fax: +48 22 572 09 85

e-mail address: afilipek@wum.edu.pl (A.Filipek)

ORCID - Agnieszka Filipek: 0000-0002-1351-3687

Table 1s. Frequency analysis of quantitative variables. Classification due to haptoglobin type.

**Variable**	**Hp 1-1 (N = 9)**		**Hp 2-1 (N = 37)**		**Hp 2-2 (N = 48)**		**$H_{(df = 2)}$**	**P-value[1]**
	**Mdn**	**IQR**	**Mdn**	**IQR**	**Mdn**	**IQR**		
Age [years]	61.0	8.0	59.0	11.0	61.5	9.0	2.680	0.262
BMI [kg/m2]	34.0	9.0	34.0	6.0	32.0	9.0	0.941	0.625
Waist [cm]	112.0	18.0	116.0	18.0	112.5	18.5	1.555	0.460
Duration of Diabetes [years]	18.0	12.0	12.0	9.0	10.0	10.5	1.350	0.509
Fasting glucose [mg/dl]	180.0	40.0	160.0	50.0	165.0	60.0	3.472	0.176
Glycemia 2h after a meal [mg/dl]	210.0	100.0	200.0	60.0	250.0	100.0	9.537	0.009
HbA1c [%]	9.4	2.1	9.0	1.8	9.4	2.4	1.092	0.579
CRP [mg/l]	2.6	1.7	2.4	2.8	2.2	3.4	0.197	0.906
Ca [mmol/L]	2.4	0.2	2.4	0.1	2.4	0.1	0.180	0.914
Phosphorus [mg/dl]	3.7	1.0	3.5	0.6	3.8	0.8	1.694	0.429
Vitamin D [ng/ml]	17.1	8.2	16.4	8.6	17.9	9.1	0.287	0.867
Daily dose of metformin [mg]	1850.0	1200.0	2550.0	1500.0	2550.0	1300.0	1.517	0.468
Leukocytes [tys./ul]	6.9	2.4	6.6	1.7	7.9	2.2	5.799	0.055
Neutrophils [tys./ul]	4.0	1.2	3.7	0.9	4.3	1.7	5.830	0.054
Neutrophils [%]	55.7	9.3	54.7	10.7	57.1	9.8	1.401	0.496
Lymphocytes [k/ul]	2.4	1.1	2.3	1.1	2.3	0.9	2.196	0.334
Lymphocytes [%]	32.4	6.2	31.4	10.6	30.3	7.6	2.612	0.271
Haemoglobin [g/dl]	13.4	1.1	14.9	2.0	14.3	2.0	4.187	0.123
Erythrocytes [M/ul]	4.8	0.7	4.9	0.6	4.8	0.7	1.972	0.373
Haematocrit [%]	40.7	3.3	44.6	7.2	43.1	4.9	3.193	0.203
MCV [fl]	90.4	7.9	91.1	6.8	89.8	5.5	1.408	0.495
Platelets [k/ul]	241.0	63.0	233.0	81.0	236.0	74.0	0.346	0.841
RDW-SD	41.3	2.4	41.7	5.4	43.4	3.6	3.586	0.166
RDW [%]	12.6	1.5	12.6	1.0	13.2	0.9	4.174	0.124
IL-10 [pg/ml]	236.4	933.7	30.4	35.0	35.8	58.5	24.258	0.000
p53 [U/ml]	39.1	7.1	57.5	64.6	81.6	91.9	13.634	0.001
sCD 163 [ng/ml]	287.4	99.7	130.4	48.3	114.0	48.6	24.768	0.000
HMGB1 [pg/ml]	1024.5	244.5	3786.5	903.6	3858.2	840.6	24.411	0.000

Mdn – median, IQR –interquartile range, df –degrees of freedom

[1] Kruskal–Wallis H test

Table 2s. Frequency analysis of quantitative variables. Parametric analysis with the t-student test Classification due to cardiovascular complications.

Variable	Group without complications (N = 60)		Group with complications (N = 34)		$t_{(df = 92)}$	P-value[2]
	**M**	**SD**	**M**	**SD**		
Age[years]	59.08	7.60	61.88	6.97	-1.767	0.081
Waist [cm]	111.32	15.23	116.03	11.58	-1.564	0.121
HbA1c [%]	9.35	1.62	9.41	1.39	-0.188	0.851
Phosphorus [mg/dl]	3.63	0.65	3.79	0.58	-1.186	0.239
Neutrophils [%]	56.21	7.83	55.59	6.79	0.390	0,698
Lymphocytes [k/ul]	2.31	0.69	2.38	0.57	-0.496	0.621
MCV [fl]	89.81	3.78	91.21	4.62	-1.599	0.113
Platelets [k/ul]	239.77	60.17	237.32	50.14	0.200	0.842

M –mean, SD –standard deviation , df –degrees of freedom

[2] Student's t-test
